# Influence of Polymethylsilsesquioxane Content to the Thermal Stability of Meta-Aramid Fiber Insulation Paper

**DOI:** 10.3390/ma11112317

**Published:** 2018-11-19

**Authors:** Wei Zheng, Jufang Xie, Jingwen Zhang, Chao Tang, Zhongyong Zhao

**Affiliations:** College of Engineering and Technology, Southwest University Chongqing, Chongqing 400715, China; zhengwei_0406@163.com (W.Z.); m18227592296_1@163.com (J.Z.); zhaozhongyong@cqu.edu.cn (Z.Z.)

**Keywords:** polymethylsilsesquioxane, meta-aramid fiber insulation paper, thermal stability, mechanical properties

## Abstract

Polymethylsilsesquioxane (PMSQ) nanoparticles with mass percentages of 0, 2.5, 5.0, 7.2, 9.4 wt %, respectively, were constructed by molecular dynamics methods in this paper. Composite molecular models were established using PMSQ and MPIA (poly-metaphenylene isophthalamide) fiber. The influence of different PMSQ contents on the thermal stability of meta-aramid insulation paper was analyzed from the parameters of mechanical property, interaction energy, and mean square displacement. The results showed that the trend of mechanical properties decreased with the increase of PMSQ content. When the PMSQ content was 2.5 wt %, the mechanical properties of the composited model were the best, which was about 24% higher than that of the unmodified model. From an intermolecular bonding and nonbonding point of view, the energy parameters of composite model with the 2.5 wt % content was better than those of the composite model with other contents. Therefore, it is considered that MPIA can interact better with the 2.5 wt % content PMSQ composite model. When the PMSQ content is 2.5 wt %, the overall chain movement in the composite model is slower than that of the unmodified model, which can effectively inhibit the diffusion movement of the MPIA chain. In general, the thermal stability of composite molecular models MPIA and PMSQ (2.5 wt %) was better improved.

## 1. Introduction

With the continuous improvement of the voltage level of transmission lines in China, higher quality requirements have been imposed on the insulation performance of internal transformer components. Cellulose insulation paper has been widely used in the insulation of power transformers due to its good insulation and mechanical properties, easy harvesting, simple manufacturing process, and electrical insulation system with good transformer-oil performance [1,2]. Cellulose insulation performance is not optimal in electrical insulation materials, despite its wide range of raw materials and easy manufacturing process. Meta-aramid fiber insulation paper is widely used in the military, communications, transportation, electrical, and other industries because of its special structure and excellent mechanical, chemical, electrical, and physical properties. Especially in the electrical field, meta-aramid has gradually become a new type of insulation paper instead of traditional cellulose insulation paper. At the same time, research on the modification of organic polymers by using inorganic nanoparticles has been extensively carried out. The modified materials have the characteristics of easy processing, low cost, and light weight. At the same time, its heat resistance and mechanical properties have also been greatly improved.

Polyhedral oligomeric silsesquioxan (POSS) is a kind of compound with a cage, trapezoidal, and bridge structure. It is composed of a Si-O-Si bond inside the molecule and links various organic groups on the external silicon atom. Its monomers are composite of organic and inorganic [3,4,5,6]. This special composition of molecular structure gives it many excellent properties, such as high- and low-temperature resistance, electrical insulation, hydrophobicity, flame retardancy, nontoxicity, corrosion, and physiology. Therefore, one or more active groups were attached to Si atoms on POSS, and POSS derivatives with special properties were synthesized by chemical methods [4,6,7,8]. The new derivatives not only had the properties of the molecular structure itself, but also had some of the properties of the added functional groups. They were introduced to organic polymers through nanoenhancement between nanoparticles and a polymer organic matrix by chemical or physical means. The modified material has the dual advantages of inorganic nanomaterials and organic polymer materials. POSS can improve the thermal stability [4,6,9,10,11,12], mechanical properties [4,6,12,13,14], hydrophobicity [15,16,17], flame retardancy [18,19,20], and dielectric properties [21,22,23] of the polymer at the molecular level; this modification method has been widely studied by researchers.

Based on the above studies, polymethylsilsesquioxane [4,6] was used to modify aramid insulation paper, and the blending model of polymethylsilsesquioxane and meta-aramid insulation paper was established by means of molecular mechanics and dynamics. The changes of the macro- and microparameters of polymethylsilsesquioxane-modified meta-aramid insulating paper were analyzed. In order to provide theoretical support for the further study of nanomodified insulation paper, the intrinsic mechanism of improving the thermal stability of meta-aramid fiber insulation paper by doping polymethylsilsesquioxane was explored.

## 2. Model Construction

**Figure 1:**
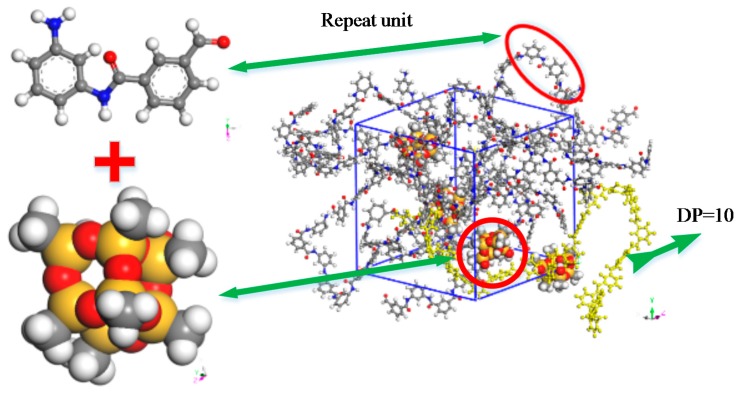
Schematic diagram of composite model.

The degree of polymerization (DP) of the polymer is quite large. The DP of the cellulose is about 800–1000 [24], while the molecular number of the poly-metaphenylene isophthalamide (MPIA) fiber is 60,000–900,000 [25]. Based on local and international research results, and the experience of our team [26,27,28,29], five composited models of polymethylsilsesquioxane (PMSQ) and MPIA fiber with mass percentages of 0, 2.5, 5.0, 7.2, and 9.4 wt % were constructed. The simulation was carried out on Materials Studio (Accelrys, San Diego, CA, USA) software, and the model was built with Amorphous Cell (AC). The MPIA was constructed with a DP of 10, a chain number of 8, and an initial density of 1.0 g/cm^3^. Geometric optimization, annealing, and dynamics simulation of the built model were all carried out under a Forcite module, and references [27,28,29] were referred to when setting the parameters. The reason for choosing the COMPASS force field [28,30,31] is that the error between the simulated and experimental data under the COMPASS force field is within the allowable range, indicating the credibility of the force field [32,33]. At the same time, the density of the polymer under the COMPASS field is closer to the real density, which is more conducive to the molecular-dynamics simulation [28]. The normal operating temperature of the transformer is 313–333 K, and the local temperature can rapidly reach 383 K. Meta-aramid fiber insulation paper is the main insulating material of the transformer, and conventional thermal aging temperature is 343–423 K [34]. Therefore, the simulated temperature range was 343–423 K, and the temperature interval is 20 K—a total of five models. The schematic diagram of the composite model is shown in Figure 1.

## 3. Parameter Calculation and Result Analysis

### 3.1. Mechanical Property

It is known from elastic mechanics that the general relationship between the stress and strain of a solid material can be expressed by generalized Hooke’s law [35].

(1) $$\left[\begin{array}{c} \sigma_{x}\\ \sigma_{y}\\ \sigma_{z}\\ \tau_{yz}\\ \tau_{zx}\\ \tau_{xy} \end{array}\right]=\left[\begin{array}{cccccc} C_{11} & C_{12} & C_{13} & C_{14} & C_{15} & C_{16}\\ C_{21} & C_{22} & C_{23} & C_{24} & C_{25} & C_{26}\\ C_{31} & C_{32} & C_{33} & C_{34} & C_{35} & C_{36}\\ C_{41} & C_{42} & C_{43} & C_{44} & C_{45} & C_{46}\\ C_{51} & C_{52} & C_{53} & C_{54} & C_{55} & C_{56}\\ C_{61} & C_{62} & C_{63} & C_{64} & C_{65} & C_{66} \end{array}\right]\left[\begin{array}{c} \varepsilon_{x}\\ \varepsilon_{y}\\ \varepsilon_{z}\\ \gamma_{yz}\\ \gamma_{zx}\\ \gamma_{xy} \end{array}\right]$$

where $C_{ij}$ is a 6 × 6 elastic coefficient matrix element. All mechanical parameters of the material can be derived from its elastic coefficient matrix, where σ is stress, *ε* is strain, *τ* is shear stress, and *γ* is shear strain. According to elasticity, the mechanical parameters of the materials can be calculated, including the bulk modulus (*K*), shear modulus (*G*), elastic modulus (*E*), Poisson’s ratio (V), and Cauchy pressure (C_12_–C_44_). These parameters can be used to represent the different mechanical properties of materials [35].

#### 3.1.1. Elastic Modulus (*E*)

**Figure 2:**
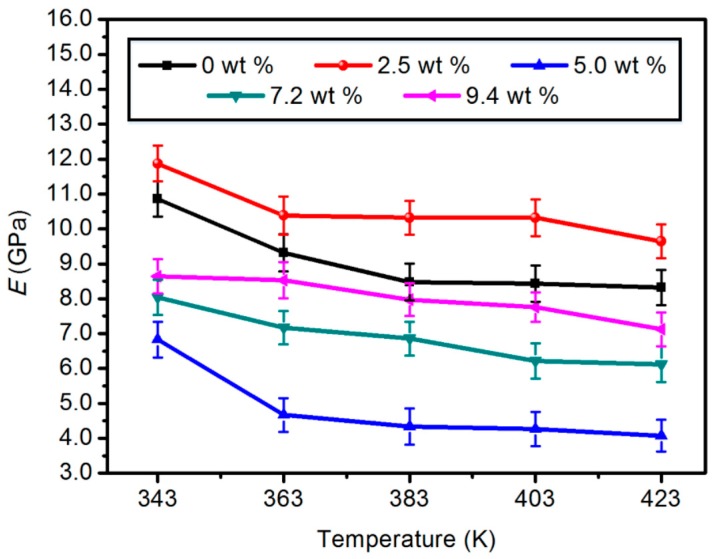
Elastic modulus of five different content composite models.

This refers to the ratio constant of the longitudinal stress on the longitudinal strain acting on the material in the range of elastic deformation (within the proportional limit). It also often refers to the ratio of stresses, such as tensile, compressive, curved, twisted, and sheared, to the corresponding strain produced by the material. The greater the ratio, the stronger the rigidity and resistance to deformation of the material. As shown in Figure 2, the modulus of elasticity of both unmodified and modified models decreased with the increase of the operating temperature. However, it is obvious that the elastic modulus value of the modified model with a content of 2.5 wt % always remained the highest with increasing temperature. Among the selected temperatures, the value of the modified model of 2.5 wt % was 9.4%, 11.6%, 21.7%, 22.3%, and 15.9% higher than that of the unmodified model, respectively. Therefore, the elastic modulus of the modified model with a content of 2.5 wt % had significant improvement. Meanwhile, the value of the modified model with 5.0, 7.2, and 9.4 wt % content is smaller than that of the unmodified model. So, from the perspective of elastic modulus, it can be known that the modified model with a content of 2.5 wt % is relatively optimal.

#### 3.1.2. Shear Modulus (*G*)

**Figure 3:**
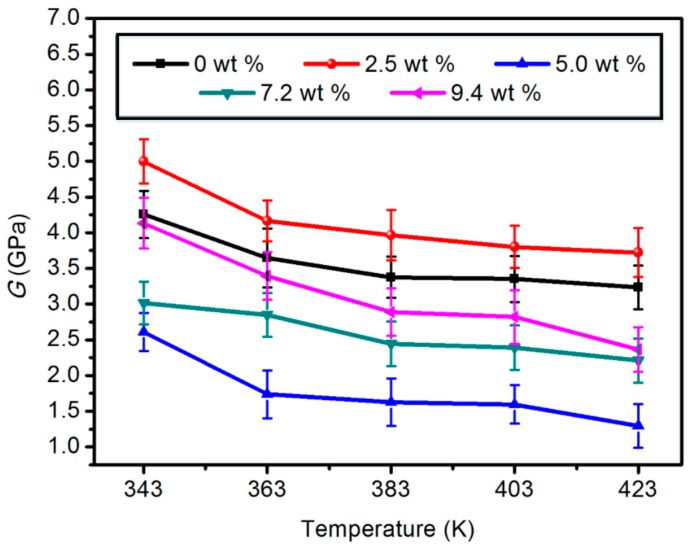
Shear modulus of five different content composite models.

This is the ratio of shear stress to strain. The larger the value, the stronger the ability of the material to resist shear stress, and damage is not likely to occur under external stress. As shown in Figure 3, the shear modulus of the modified model with 2.5 wt % content was the highest among the five modified models, which is similar to that of the elastic modulus. The values of the shear modulus in the modified model with 5.0, 7.2, and 9.4 wt % content were smaller than those of the unmodified model. It can be seen that, with the increase of temperature, the shear modulus of the modified model with 2.5 wt % content was relatively optimal, which can enhance the shear stress resistance of the material.

#### 3.1.3. Bulk Modulus (*K*)

**Figure 4:**
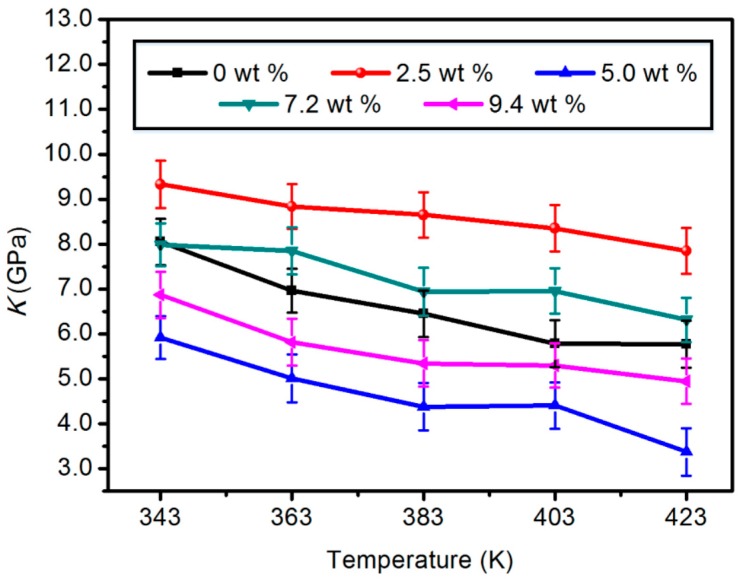
Bulk modulus of five different content composite models.

This can be used to describe the elasticity of homogeneous isotropic solids or the force per unit area, indicating incompressibility. The greater the value, the stronger the incompressibility of the materials. It can be seen from Figure 4 that the bulk modulus of several composited models shows a downward trend with the increase of the operating temperature. Similar to the previous parameters, the 2.5 wt % modified model is relatively optimal in terms of bulk modulus.

#### 3.1.4. Poisson’s Ratio (V) and Cauchy Pressure (C_12_–C_44_)

From Table 1, it can be seen that Poisson’s ratio only reflects the mutual influence of material deformation in different directions, and has nothing to do with the degree of deformation. In view of this, the Poisson’s ratio (average value) of the modified model with 2.5 wt % content is the smallest in the five composite models, and it is considered that the interaction between the deformation of materials in different directions is relatively small. Cauchy pressure reflects the ductility of the material. When the Poisson’s ratio is positive, the larger the value and the better the ductility of the material are. The negative value indicates that the material is brittle.

Table 2 shows that the unmodified model and modified model were both positive, indicating that aramid fiber itself has a certain ductility, through the addition of nanoparticles, increased the ductility of materials. The more significant changes were the contents of 2.5 and 7.2 wt %.

After analyzing the elastic, shear, and bulk modulus, Poisson’s ratio, and Cauchy pressure, the mechanical properties of the unmodified model show a downward trend with the increase of temperature. The mechanical properties of the modified model with a content of 2.5 wt % were further improved compared with the unmodified model. On the one hand, the meta-aramid is a linear macromolecule composed of a phthalamide groups interconnected with meta-phenyl groups. A large number of hydrogen bonds are formed between the phthalamide groups of adjacent molecular chains, and hydrogen bonds are arranged into three-dimensional structures in planes. Therefore, strong hydrogen bonding gives it a stable chemical structure, excellent mechanical properties, thermal stability, and electrical insulation properties [36,37]. On the other hand, adding a lower content of PMSQ provides greater spatial structure to the composited model. Moreover, the silicon oxide chain composed of Si-O bonds with long bond length, large bond angle, and high bond energy has low viscous-flow activation energy. At the same time, due to the mutual compensation of dπ-pπ between the Si-O bond and the compensation of the Si-O dipoles [38], the voids between molecules are reduced, and intermolecular hydrogen bonds are easier to form. Therefore, the mechanical properties and thermal stability of the material are improved [39].

### 3.2. Interaction Energy

The strength of the interaction between substances is calculated by the intermolecular interaction energy of the total energy of each system in a stable configuration. The interaction of the composite model in Reference [40] can be expressed by Equation (2):

(2) $$E_{interaction}=E_{total}-(E_{nano}+E_{polymer})$$

where $E_{interaction}$ represents the total energy of the entire simulation system, $E_{nano}$ represents the energy of the nanoparticles when they exist alone in the system, and $E_{polymer}$ represents the energy of the polymer when it exists alone in the system. If $E_{interaction}$ were negative, the nanoparticles were more likely to bind to the polymer, the binding process was exothermic, and the entire system was mutually reinforcing.

From the data in Table 3, we can see that the modified models with a content of 5.0, 7.2 and 9.4 wt % were not significantly interactive for the entire composite model. That is to say, the bond between the PMIA and the PMSQ molecule was not bonded to the bond, and the whole simulation process can be considered as a process in which the two substances independently absorb heat, although the energy of the two models is generally balanced. In fact, the physical and chemical changes that occur between the intermolecular bonding and nonbonding do not produce enough bonding energy to make a good bond between the PMIA and PMSQ molecules, thus exhibiting that the interaction of the two substances is not obvious, and the effect of blending modification is not good [41]. The modified models with 2.5 wt % content played an important role in promoting the whole composite model, and the effect of temperature was not obvious. According to the bonding effect and nonbonding action, it can be shown that the modified model with 2.5 wt % content was relatively best.

Table 4 shows that both the bonding and nonbonding energy values of the modified model with a content of 2.5 wt % were higher than those of the unmodified model. The reason is the bonding and nonbonding effect, and bonding effects mainly include bond expansion, bending of the bond angle, and twisting of the dihedral angle, while nonbonding effects mainly include Van der Waals, electrostatic, and Coulomb forces [42]. These are mainly due to the lower mobility of PMIA chains, enhanced Van der Waals force between particles, and the physical interaction between PMSQ molecules and PMIA polymer chains [43]. The adjacent PMIA chains show strong interactions. At the same time, PMSQ itself has a long bond length, large bond angle, and high bond energy. The silicon–oxygen chain composed of Si-O bonds is very soft. Its viscous-flow activation energy is low. The Coulomb interaction becomes larger and the strong bond-forming and nonbonding interactions make the amino and aldehyde groups on the PMIA chain bond with more groups, such as methyl and hydroxyl groups on the upper surface of PMSQ, to form new chemical bonds. The energy is released during the formation of new chemical bonds, while making the two materials have better compatibility during the mixing as well, thereby reducing the interaction energy of the entire composite model.

### 3.3. Mean Square Displacement

In the molecular dynamics method, each atom starts from the initial position and continuously performs thermal Brownian motion. The position of each atom is different at every moment. Mean square displacement (MSD) is the average value of particle displacement, which was often used to reflect the motion of particles. The relation between MSD and time was expressed by Reference [44], as shown in Equation (3):

(3) $$MSD\left(\Delta t\right)=\langle {\left|r_{i}\left(t_{o}+\Delta t\right)-r_{i}\left(t_{o}\right)\right|}^{2}\rangle$$

The chain movement of polymers has great influence on the thermal stability and mechanical properties of materials. The MSD can be used to characterize the chain motion of the polymer. The steeper the slope of the time curve with the polymer chain is, the worse the thermal stability of the polymer. The curve of the same trend can be directly judged according to the value. Therefore, the larger the MSD value is, the more intense the chain motion.

**Figure 5:**
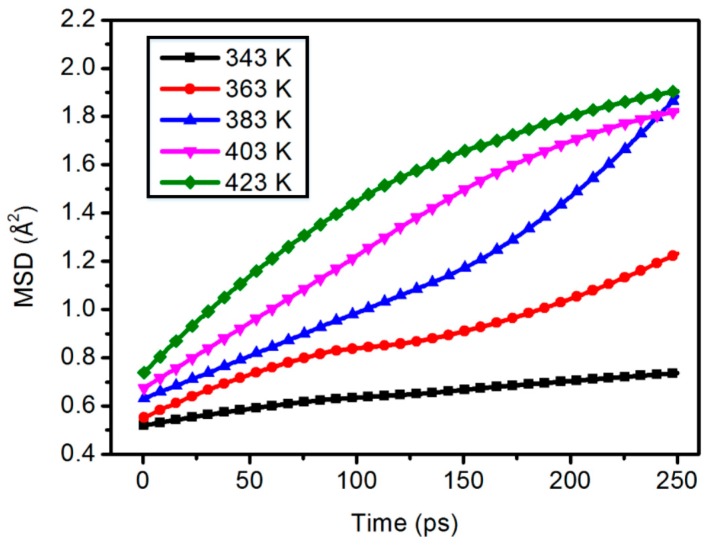
Chain motion of the 0 wt % content composite model.

**Figure 6:**
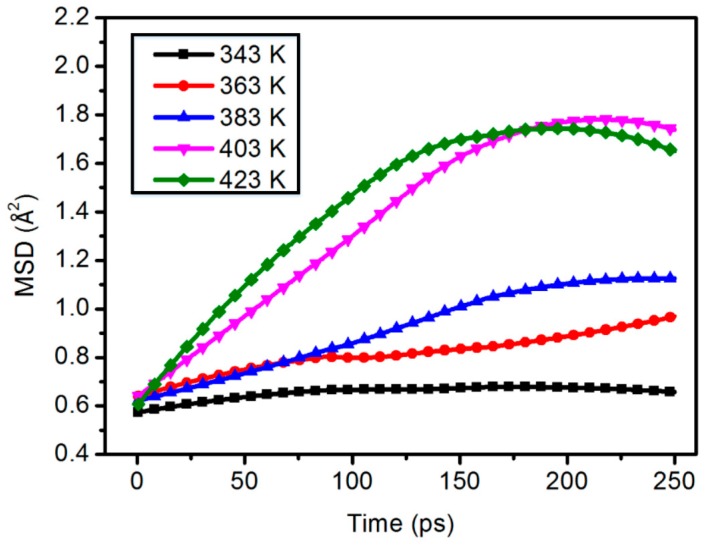
Chain motion of the 2.5 wt % content composite model.

Figure 5 and Figure 6 show the average shift of all atoms and the kinematics of polymer chains. The molecular chain motion of the polymer shows good linear change with the increase of temperature.

**Figure 7:**
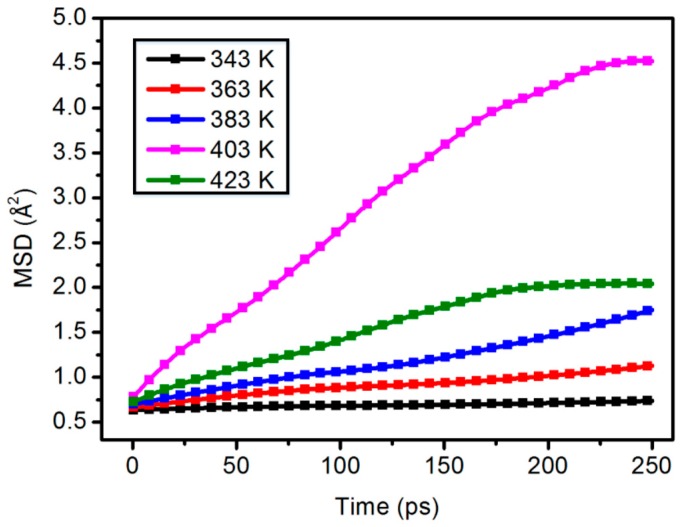
Chain motion of the 5.0 wt % content composite model.

As shown in Figure 7, at the same content, the molecular chain motion of the polymer shows a good linear change with the increase of temperature. But in the content of 5.0 wt %, the MSD curve of 423 K is lower than 403 K.

**Figure 8:**
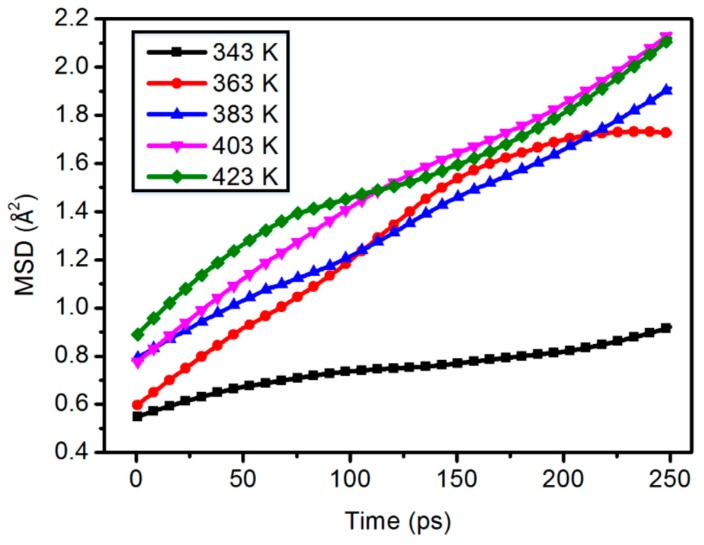
Chain motion of the 7.2 wt % content composite model.

As shown in Figure 8, as a whole, the molecular chain motion and temperature show a good positive correlation. However, in the composite model of 7.2 wt %, the MSD curves of 423 K and 403 K are basically the same after 100 ps.

**Figure 9:**
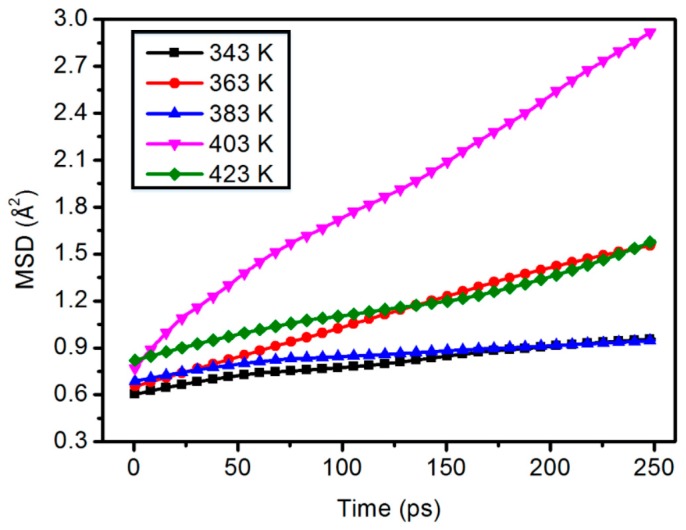
Chain motion of the 9.4 wt % content composite model.

In Figure 9, it can be clearly seen that the MSD curves at 343 and 363, 383, and 403 K are relatively close in the composite model of 9.4 wt %.

The modified-model MSD value was relatively flat when the operating temperature was low, indicating that the molecular chain moves slowly. When the operating temperature was high, the curve became steeper. The slope was also larger, indicating that the molecular chain movement was more intense [45]. With the increase of the simulation time, the difference of MSD between the curve with higher temperature and the curve with lower temperature is larger, which indicates that the molecular chain is more sensitive to temperature. At the same time, with the increase of PMSQ mass fraction at the same operating temperature, the mobility of polymer molecular chains also showed a nonlinear trend. From the overall trend, the MSD value showed the following trends: 2.5 wt % < 5.0 wt % < 0 wt % < 9.4 wt % < 7.2 wt %. Because the content was low, the MPIA chain occupied a large space that reduced the mobility of PMSQ [46]. Meanwhile, PMSQ molecules exhibited nanocrystalline phenomena and formed typical clusters due to their own interactions, weakening their own mobility [47]; then, the mobility of PMSQ decreases. This further demonstrates that the 2.5 wt % by weight PMSQ molecule had the effect of suppressing the MPIA chain kinetic strength of the MPIA fiber.

## 4. Conclusions

In this paper, molecular-dynamics methods were used to study different amounts of PMSQ for the modification of MPIA fiber insulation paper. The influence of PMSQ content on the thermal stability of meta-aramid fiber insulation paper was analyzed from the parameters of mechanical property, interaction energy, and mean square displacement. Compared with other models, the modified model with a PMSQ content of 2.5 wt % had enhanced deformation resistance, strengthened incompressibility, promoted resistance to shear stress, and improved reinforcement and ductility. Overall mechanical properties were especially improved by about 24% compared to the unmodified model. The interaction between molecules mainly depends on bonding and nonbonding. The modified model with 2.5 wt % content acts as a more active role than that with other contents, which makes the nanoparticles better combine with the aramid fiber, releases energy, and reduces the energy of the composite model. The MSD of MPIA in the modified composite model with 2.5 wt % content was weaker than that of MPIA in the unmodified composite model, indicating that the PMSQ molecule can be relatively easily dispersed in MPIA molecules and inhibit the molecular chain motion of MPIA fiber.

Generally speaking, when the content of PMSQ is 2.5 wt %, the thermal stability of the composite model of PMSQ and MPIA is quite better than that of the unmodified model, which provides theoretical support for the further study of nanomodified insulation paper.

## Figures and Tables

**Table 1:** Poisson’s ratio of five different composite models (average value).

Mass Percentage (wt %)	Poisson’s Ratio (V)
0 wt %	0.2701
2.5 wt %	0.2567
5.0 wt %	0.3300
7.2 wt %	0.3103
9.4 wt %	0.2945

**Table 2:** Cauchy pressure of five different composite models (average value).

Mass Percentage (wt %)	Cauchy Pressure (C_12_–C_44_)
0 wt %	0.7934
2.5 wt %	1.4826
5.0 wt %	1.1685
7.2 wt %	1.9970
9.4 wt %	1.3886

**Table 3:** Interaction energies of different polymethylsilsesquioxane (PMSQ) contents at different temperatures.

Temperature (K)	Mass Percentage (wt %)	$E_{interaction}$ (kcal/mol)	$E_{total}$ (kcal/mol)	$E_{nano}$ (kcal/mol)	$E_{polymer}$ (kcal/mol)
343	2.5 wt %	−60.30	22,758.54	−798.98	23,617.82
	5.0 wt %	657.29	18,530.88	−1018.68	18,892.27
	7.2 wt %	330.34	18,925.28	−2362.19	20,957.13
	9.4 wt %	301.74	244,53.84	−2962.59	27,114.69
363	2.5 wt %	−57.90	228,10.24	−783.35	23,651.50
	5.0 wt %	695.99	18,631.31	−1028.56	18,963.88
	7.2 wt %	319.10	19,168.66	−2372.55	21,222.11
	9.4 wt %	280.73	24,646.37	−2945.34	27,310.98
383	2.5 wt %	−67.12	22,971.43	−774.72	23,813.27
	5.0 wt %	673.10	18,780.74	−999.14	19,106.77
	7.2 wt %	301.50	19,412.03	−2340.20	21,450.73
	9.4 wt %	294.69	24,683.29	−2960.78	27,349.37
403	2.5 wt %	−62.94	23,014.71	−779.71	23,857.36
	5.0 wt %	693.37	18,896.53	−1059.95	19,263.11
	7.2 wt %	278.32	19,595.41	−2368.89	21,685.98
	9.4 wt %	293.77	25,037.45	−2899.78	27,643.45
423	2.5 wt %	−56.95	23,184.02	−778.17	24,019.14
	5.0 wt %	667.98	19,090.43	−982.06	19,404.52
	7.2 wt %	275.66	19,938.79	−2342.38	22,005.51
	9.4 wt %	266.30	24,954.38	−2923.85	27,611.94

**Table 4:** Energy values of bonding and nonbonding interactions at different temperatures.

Temperature (K)	Energy	0 wt % (kcal/mol)	2.5 wt % (kcal/mol)
343	Valence energy	11,898.25	13,565.77
	Nonbond energy	6050.19	6498.12
363	Valence energy	11,748.40	13,534.63
	Nonbond energy	6227.78	6635.92
383	Valence energy	12,126.83	13,835.83
	Nonbond energy	6113.74	6496.96
403	Valence energy	12,127.38	13,927.94
	Nonbond energy	6403.19	6626.21
423	Valence energy	12,358.77	14,136.22
	Nonbond energy	6259.24	6399.32
